# COVID-19 and physical inactivity: Teetering on the edge of a deadlier pandemic?

**DOI:** 10.7189/jogh.11.03031

**Published:** 2021-02-11

**Authors:** Sherief Ghozy, Abdelaziz Abdelaal, Jaffer Shah, Kate Elizabeth Parker, Sheikh Mohammed Shariful Islam

**Affiliations:** 1 Faculty of Medicine, Mansoura University, Mansoura, Egypt; 2 Faculty of Medicine, Tanta University, Tanta, Egypt; 3 Drexel University College of Medicine, Philadelphia, Pennsylvania, USA; 4 Institute for Physical Activity and Nutrition (IPAN), School of Exercise and Nutrition Sciences, Deakin University, Geelong, Australia

On March 11, 2020, the World Health Organization (WHO) declared COVID-19 as a global pandemic. To limit its spread, most countries recommended social distancing, quarantine and self-isolation, and closure of schools and most businesses. This has resulted in a substantial negative impact on human society regarding health, economy, and lifestyle. In turn, health care policymakers focused on diagnosis and management of COVID-19, as well as the development of effective vaccines. However, the long-term effects of the COVID-19 pandemic on physical activity (PA) and sedentary behaviour (SB) of the world’s population, secondary to quarantine, with their consequent burden on global health, have not been given their required attention. We aim to highlight the need for all physicians to include a prescription of increasing PA and reducing SB for their patients during and following COVID-19, particularly those with known comorbidities.

Physical activity is an essential indicator of health status and has been highlighted as critically important for mitigating the harms of the coronavirus disease-2019 on health and well-being. Quarantine and social distancing measures are impacting the ways in which people can be active and many studies have shown a significant decrease in PA participation concurrent with an increase in SB compared to pre-pandemic levels. For example, Tison et al. [1] conducted a study to determine the daily step count, an indicator of PA, among 455 404 individuals from 187 different countries. The mean daily step count reduced by 5.5% within the first 10 days of pandemic declaration and by 27.3% within 30 days. This figure is expected to worsen as time goes by. Meyer et al. studied changes in PA and screen-based SB in 3052 US adults from all 50 states. They found a clinically meaningful reduction in PA and increase in sitting and screen time (common indicator of SB) among those in self-isolation, with decreases in PA (-32%) seen to be worst among those who were previously active [2]. These lifestyle changes were also observed in children. For example, studies conducted with large samples of children and adolescents in the US [3] and Germany [4] saw a reduction in PA and greater engagement in SB during the pandemic compared to earlier periods. These studies highlight dramatic negative changes in PA and SB, which is concerning given the considerable health risks associated with insufficient PA and excessive SB.

It is well-documented that regular engagement in physical activities enhances the effectiveness of the immune system [5], which would affect the severity of COVID-19. Therefore, the noticeable increase in physical inactivity and excess sedentary time could potentially lead to the deterioration of infected cases and further promote the spread of the disease. Furthermore, there are serious concerns that the documented short-term deteriorations in PA and increases in SB may become permanently fixed. This may subsequently increase the risk of obesity, diabetes, and cardiovascular diseases, as well as exacerbate the severity of existing conditions [6].

Obesity and PA are linked, with an almost 4-fold increase in the risk of obesity in physically inactive individuals [7]. Obese individuals are also disproportionately affected by COVID-19 infection [8]. It is reported that obesity increases the risk of COVID-19 infection by 46%, hospitalizations by 113%, ICU admission by 74% and mortality by 48% [8]. Moreover, obesity is linked with major cardiometabolic disorders, including hypertension, diabetes, and other comorbidities. These comorbidities have been reported to be associated with worse prognosis and serious adverse events in patients with COVID-19, as hypertension and diabetes were documented in 13.34% and 9.65% of total COVID-19 cases and 47.90% and 24.89% of mortality cases, respectively [9]. These statistics warn us about the current situation and impact of COVID-19 on obesity, secondary to physical inactivity and SB, with the potential of a ‘second wave’ that would be even worse compared to the early pandemic statistics. Therefore, an increase in PA and reduction in SB should be advocated to avoid any permanent changes in behavior and associated ill health lasting beyond the quarantine duration. For instance, physicians could inform their patients on ways to remain active during times of social distancing and isolation by performing outdoor low-to-moderate intensity PA daily, incorporate stretching and breathing deeply, break up sitting times with PA, standing up, and stretching and avoiding inactivity throughout the day [10]. During the COVID-19 pandemic, there have been substantial increases in the use of remote and telehealth services by physicians. These online services also offer an ideal platform whereby physicians can conduct PA and SB monitoring and adjunct behaviour change approaches to patients with inactive lifestyles when access to in-person services are unavailable. PA promotion interventions can be implemented in primary health care settings. Primary care professionals are well placed to support positive changes in PA and SB since they represent a trusted source of health advice. However, physicians may not be fully equipped to provide appropriate PA counselling, as it is not comprehensively covered in the medical curriculum [11], and counselling time with patients is limited allowing only a few issues to be addressed in a single visit. Thus, there may be limited capacity to assess current activity levels, determine readiness for change or prescribe PA. Despite these limitations, PA promotion interventions in primary health care settings have shown short and long-term effectiveness and should be enforced by primary care commissioners and local government health departments [12,13].

In addition to intrapersonal support mechanisms such as physicians prescribing and supporting their patients to be active, population level initiatives are also required. As proposed by socio-ecological models, lifestyle behaviours are influenced by a broad range of factors and as such, efforts to support and maintain PA require comprehensive and synergistic efforts by policymakers, educators, community groups, health care professionals, and the general population [14]. Such initiatives may include policymakers limiting or prohibiting vehicle traffic at certain times to encourage active travel, sporting organisations and educators harnessing social media and online platforms to promote safe, age-appropriate home-based exercises or family activity challenges, and community groups providing information on local walking routes and green spaces to encourage neighbourhood activity. For children and adolescents, enjoyment is a key factor so, creative methods like walking a dog, TikTok dancing, online PA classes, gaming-based PA, and scavenger hunts; should be used. At the community level, parents should be educated about how to maintain the PA of their children at home along with showing children-oriented educational videos at schools and community centres.

**Figure:**
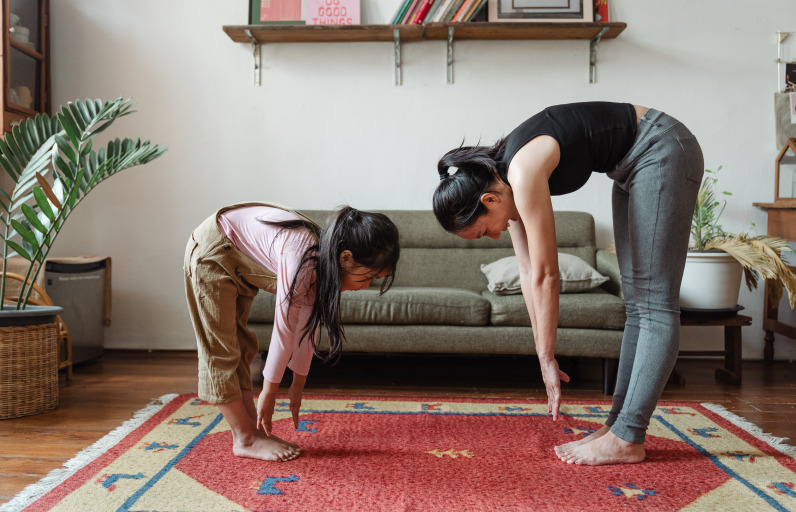
Photo: From https://unsplash.com/.

Global initiatives for a COVID-19 vaccine are showing promise. However, a major concern is that COVID-19 vaccines would be less effective in obese individuals compared to the general population [8], given the fact that obesity is linked to suboptimal vaccine responses for a number of diseases, such as influenza [15] and tetanus [16]. Thus, in addition to population wide policy and strategy implementation, it is critical that physicians include a prescription of PA and a reduction in SB for prevention and management of obesity and associated comorbidities, not only during the pandemic and initial vaccination phases, but also in the long-term.

## References

[1] Tison GH Avram R Kuhar P Abreau S Marcus GM Pletcher MJ Worldwide Effect of COVID-19 on Physical Activity: A Descriptive Study. Ann Intern Med. 2020;173:767-70. 10.7326/M20-2665 32598162 PMC7384265

[2] Meyer J McDowell C Lansing J Brower C Smith L Tully M Changes in Physical Activity and Sedentary Behavior in Response to COVID-19 and Their Associations with Mental Health in 3052 US Adults. Int J Environ Res Public Health. 2020;17:6469. 10.3390/ijerph17186469 32899495 PMC7559240

[3] Dunton GF Do B Wang SD Early effects of the COVID-19 pandemic on physical activity and sedentary behavior in children living in the U.S. BMC Public Health. 2020;20:1351. 10.1186/s12889-020-09429-3 32887592 PMC7472405

[4] Schmidt SCE Anedda B Burchartz A Eichsteller A Kolb S Nigg C Physical activity and screen time of children and adolescents before and during the COVID-19 lockdown in Germany: a natural experiment. Sci Rep. 2020;10:21780. 10.1038/s41598-020-78438-4 33311526 PMC7733438

[5] Krüger K Mooren F-C Pilat C The immunomodulatory effects of physical activity. Curr Pharm Des. 2016;22:3730-48. 10.2174/1381612822666160322145107 27000826

[6] Mattioli AV Sciomer S Cocchi C Maffei S Gallina S Quarantine during COVID-19 outbreak: Changes in diet and physical activity increase the risk of cardiovascular disease. Nutr Metab Cardiovasc Dis. 2020;30:1409-17. 10.1016/j.numecd.2020.05.020 32571612 PMC7260516

[7] Pietiläinen KH Kaprio J Borg P Plasqui G Yki-Järvinen H Kujala UM Physical inactivity and obesity: a vicious circle. Obesity (Silver Spring). 2008;16:409-14. 10.1038/oby.2007.72 18239652 PMC2249563

[8] Popkin BM Du S Green WD Beck MA Algaith T Herbst CH Individuals with obesity and COVID-19: A global perspective on the epidemiology and biological relationships. Obes Rev. 2020;21:e13128. 10.1111/obr.13128 32845580 PMC7461480

[9] Gold MS Sehayek D Gabrielli S Zhang X McCusker C Ben-Shoshan M COVID-19 and comorbidities: a systematic review and meta-analysis. Postgrad Med. 2020;132:749-55. 10.1080/00325481.2020.1786964 32573311

[10] Jurak G Morrison SA Leskošek B Kovač M Hadžić V Vodičar J Physical activity recommendations during the coronavirus disease-2019 virus outbreak. J Sport Health Sci. 2020;9:325-7. 10.1016/j.jshs.2020.05.003 32426171 PMC7229466

[11] Ward M A Survey of Physical Activity in Medical Curricula: A report of the HEPA in Health Care Settings Working Group 2015.

[12] Orrow G Kinmonth A-L Sanderson S Sutton S Effectiveness of physical activity promotion based in primary care: systematic review and meta-analysis of randomised controlled trials. BMJ. 2012;344:e1389. 10.1136/bmj.e1389 22451477 PMC3312793

[13] Sanchez A Bully P Martinez C Grandes G Effectiveness of physical activity promotion interventions in primary care: A review of reviews. Prev Med. 2015;76:S56-67. 10.1016/j.ypmed.2014.09.012 25263343

[14] Sallis J, Owen N, Fisher E. Ecological Models of Health Behavior. Health Behavior and Health Education. 2008;4.

[15] Neidich SD Green WD Rebeles J Karlsson EA Schultz-Cherry S Noah TL Increased risk of influenza among vaccinated adults who are obese. Int J Obes (Lond). 2017;41:1324-30. 10.1038/ijo.2017.131 28584297 PMC5585026

[16] Eliakim A Schwindt C Zaldivar F Casali P Cooper DM Reduced tetanus antibody titers in overweight children. Autoimmunity. 2006;39:137-41. 10.1080/08916930600597326 16698670 PMC4623573

